# The Health Behaviour of German Outpatient Caregivers in Relation to Their Working Conditions: A Qualitative Study

**DOI:** 10.3390/ijerph18115942

**Published:** 2021-06-01

**Authors:** Natascha Mojtahedzadeh, Elisabeth Rohwer, Felix Alexander Neumann, Albert Nienhaus, Matthias Augustin, Birgit-Christiane Zyriax, Volker Harth, Stefanie Mache

**Affiliations:** 1 Institute for Occupational and Maritime Medicine (ZfAM), University Medical Centre, Hamburg-Eppendorf (UKE), Seewartenstr. 10, 20459 Hamburg, Germany; n.mojtahedzadeh@uke.de (N.M.); e.rohwer@uke.de (E.R.); harth@uke.de (V.H.); 2 Midwifery Science—Health Services Research and Prevention, Institute for Health Service Research in Dermatology and Nursing (IVDP), University Medical Centre Hamburg-Eppendorf (UKE), Martinistr. 52, 20246 Hamburg, Germany; fe.neumann@uke.de (F.A.N.); b.zyriax@uke.de (B.-C.Z.); 3 Department of Occupational Medicine, Hazardous Substances and Public Health, Institution for Statutory Accident Insurance and Prevention in the Health and Welfare Services (BGW), Pappelallee 33/35/37, 22089 Hamburg, Germany; a.nienhaus@uke.de; 4 Institute for Health Service Research in Dermatology and Nursing (IVDP), Competence Centre for Epidemiology and Health Services Research for Healthcare Professionals (CVcare), University Medical Centre Hamburg-Eppendorf (UKE), Martinistr. 52, 20246 Hamburg, Germany; 5 Institute for Health Service Research in Dermatology and Nursing (IVDP), Competence Centre for Health Services Research in Vascular Diseases (CVvasc), University Medical Centre Hamburg-Eppendorf (UKE), Martinistr. 52, 20246 Hamburg, Germany; m.augustin@uke.de

**Keywords:** health behaviour, outpatient care, regeneration, nutrition, physical activity, stress

## Abstract

Ongoing demographic change is leading to an increasingly older society and a rising proportion of people in need of care in the German population. Therefore, the professional group of outpatient caregivers is highly relevant. Their work is characterised not only by interacting with patients in a mobile setting but also by working in shifts. Health behaviour under these specific working conditions is crucial for ensuring long-term work ability and performance. Little is known about the health behaviour of German outpatient caregivers and its potential impact on their work. The aims of the study were (1) to examine health behavioural patterns (nutrition, exercise, smoking, regeneration) of outpatient caregivers, (2) to illuminate their personal health-promoting behaviours, and (3) to identify potential work-related factors influencing their health behaviour. Fifteen problem-centred interviews were conducted with outpatient caregivers working in Northern Germany in the period January–April 2020. Interviews were analysed by using qualitative content analysis. Outpatient caregivers reported improvable nutrition and hydration, with simultaneous high coffee consumption, low physical activity, poor regeneration (breaks and sleep quality), and good personal health-promoting behaviour (e.g., back-friendly habits), although the majority were smokers. Barriers to the implementation of health-promoting behaviours were a high perception of stress due to increased workload and time pressure, while aids to better health-promoting behaviour were described as being social support and personal resources. The respondents perceived their working conditions as potentially influencing their health behaviour. On the basis of their descriptions, various practice-relevant strategies were derived. The data explore a potential need for outpatient care services to develop interventions on behavioural and structural levels that can help create healthier working conditions for their employees so these caregivers can adopt better health behaviours.

## 1. Introduction

### 1.1. Background

In Germany, a demographic change is being recorded. In the population, the group of people 60 years old or older is constantly increasing and will increase to nearly half of Germany’s total population (40%). Getting older raises the risk of being in need of care someday (see [1]). At the end of the year 2019, there were 4.1 million care-dependents in Germany. The number of individuals being cared for through outpatient care increased substantially. Comparing the years 2015 and 2017, nearly 19% more of the care-dependents in Germany were taken care of by outpatient caregivers (2019: 982,604). In 2019, there were 421,550 outpatient caregivers working in Germany. Most of them were qualified as geriatric nurses (98,976), geriatric care assistants (21,831), health and care nurses (78,129), or nursing assistants (14,822) [2]. Outpatient care is characterised by several work activities. These include body-related care measures (personal hygiene, nutrition, promotion of mobility), nursing care measures (e.g., help with orientation, organizing everyday life, or maintaining social contacts) and home nursing (administration of medication, bandage changes, injections) [3]. In the context of the constantly increasing number of outpatient care services in Germany (between 2015 and 2019, 1365 outpatient care facilities were added, for a total of 14,688) [2], the health of outpatient caregivers has become highly relevant.

By 2018, among German employees, higher rates of incapacity to work were found among the nursing professions compared with other occupational groups [4]. A high amount of work demands might discourage many young people from engaging in a job in care [5]. A shortage of skilled care workers is resulting in an increase in the number of sick leaves and early retirements in the care sector [6]. Considering employees in care work, disability days were higher in total (22.9 vs. 14.9) [7], which might trace back to bearing such strain factors as having higher amounts of work while having to maintain the same pace of work, which can lead to time pressure and performance pressure or psychosomatic illness [7,8,9,10]. According to the transactional stress model, stress can be understood as an insufficient evaluation of one’s own resources in relation to a stressor. In this way, stress is an individual reaction to stressors occurring in the person’s environment. The same stressors can therefore have different effects depending on how the person evaluates their resources for coping with stress [11,12]. Thus, for this group of employed persons, the possibility of stress perception and other negative strain reactions can be higher in care due to social conflicts with clients, among other things [11,13,14]. According to the occupational psychological stress model, which is an extension of Rohmert’s demands-strain model [15] and Lazarus and Folkman’s transactional stress model [12], condition-related and person-related stressors and risk factors as well as resources are taken into account. Based on this model, the potentially stressful situation is assessed and problem- or emotion-related coping strategies are chosen. Stress consequences therefore occur on a somatic, cognitive, emotional and/or behavioural level and can have a short- or long-term effect. Short-term consequences of stress include an increase in blood pressure and heart rate, feelings of anxiousness, anger or fatigue, and stress-influenced behaviours that lead, for example, to fluctuations in performance. Long-term consequences of stress can include stomach problems, depression, or burnout, but they can also include health-impairing behaviours such as smoking, alcohol consumption, and absenteeism [16]. Working in outpatient care is also often accompanied by time pressure and stress [9]. Thus, the resultant negative reactions to strain might lead to impairments of health [17].

Because the number of outpatient caregivers is increasing [2], it has become increasingly important to find ways of promoting healthier behaviour among individual outpatient caregivers as a way to promote the group’s overall health [18]. This approach is particularly important because the proportion of older employees in outpatient care is increasing. The age groups 30–40 years (94,499, 22.4%), 40–50 years (95,597, 22.7%), and 50–60 years (122,774, 29.1%) represent the highest proportions of employees in outpatient care and care services in Germany [2]. With increasing age, the older population of employees becomes a vulnerable group [19,20]. In addition to the possible occurrence of comorbidities, older people in general, which includes a substantial percentage of outpatient caregivers, are expected to have worse health behaviours [20,21].

Health behaviour can be divided in health-promoting (positive, e.g., healthy eating) and health-impairing (negative, e.g., consumption of tobacco, alcohol) behavioural patterns [22,23]. In the following sections, health behaviour will be divided into eating behaviour, physical activity, smoking habits, and break and recovery behaviour. Specifically, eating behaviour depicts choices of food, motives, and habits which can lead to nutrition-related problems, such as the parameters of metabolic syndrome or obesity [24]. Each movement of the body initiated by muscles and using energy describes physical activity. A lack of physical activity is a risk factor for poor health and can lead to high blood pressure and coronary heart disease [25]. Smoking behaviour, as an example of a health-impairing behavioural pattern, is widespread in Germany [26]. However, based on the data from the GEDA 2014/2015-EHIS study (“Health in Germany up-to-date”), at the time of the survey, 26.6% of German women and 35.0% of the German men had quit smoking [27]. To prevent negative reactions to stress, regular breaks should be taken [28]. Research has shown that rest breaks during working hours have clear health- and performance-promoting effects: they promote productivity, reduce fatigue and stress, and thus maintain the safety of employees at work [29,30]. Regeneration is described as a non-work-related experience that serves the well-being of individuals as well as their occupational performance [31].

Organisational structure and conditions in the care sector workplace can have a negative impact on the health behaviour of employees [32]. To promote health in the workplace, a comprehensive analysis of outpatient care workers’ health behaviour can form the basis for the design of needs-oriented, target-group-specific health-promoting measures at the behavioural and relationship level. In this way, the health, motivation, and productivity of employees can be ensured for the long term, which is also in the economic interest of care services organisations. Ultimately, employers and managers are responsible for the health of their employees [26]. With regard to the increasingly older employees who are working in outpatient care [2], working conditions must also be adapted accordingly in order to allow them to strive for a long working life in the profession [33,34,35].

### 1.2. Current State of Research

There are already several studies focusing on nurses’ health behaviour, although the majority of the participants were from the inpatient care sector (nurses, nursing students) (e.g., [36,37,38,39,40,41,42,43,44,45,46,47,48,49,50,51,52,53,54]). Some studies also examined stationary nursing staff, including geriatric nurses and educators [55] or midwives [56,57]. Table 1 depicts summarized information on the current state of the research.

#### 1.2.1. Eating Behaviour

Compared with other healthcare professionals (total *n* = 18,820), nurses (*n* = 471) showed a healthy diet in terms of their fruit/vegetable intake [53]. Nevertheless, according to previous research, unhealthy eating habits, e.g., sugary foods, are already known to be present among caregivers in the inpatient care setting. Working in shifts (early, middle, and late shift) was shown to apparently promote poor food choices by nurses (see [40]). The multi-shift system of hospital nurses also resulted in the lowest energy intake in the late shift. Carbohydrates were mostly consumed on the night shift, which had the highest stress levels, while fat was consumed more frequently on the morning shift [38]. Job demands also seem to reduce fluid intake [39]. However, nurses and midwives exhibited increased caffeine intake [56]. A Brazilian transversal study conducted with 2279 nurses (*n* = 1981 female) indicated that working hours in general were associated with eating more fried food and drinking more coffee, especially for female nurses, which eventually led some nurses to be overweight or obese [48]. Other exploratory research results suggest that inpatient nursing staff (*n* = 381) shows improvable fruit and vegetable intake [49]. In Australia, mainly the work environment of the inpatient care setting was the biggest obstacle to nurses making favourable food choices. The reasons were long working hours as well as rare opportunities for taking breaks [52]. Furthermore, perceived work stress was associated with higher calorie intake for nurses working in inpatient care [37]. Wirth et al. [55] highlighted the effects of unfavourable diets among geriatric nurses. For example, geriatric nurses had already begun to show signs of obesity during their professional training [55]. Irregular eating patterns among nurses in training are also highlighted by Binks et al. [58].

#### 1.2.2. Physical Activity

Research on registered nurses indicates that nurses were sufficiently physically active during their work activities [53,59], which has also been shown among nurses in training [58]. Exploratory research results show that of 381 Australian hospital nurses, 82% are sufficiently physically active (≥150 min/week) [49]. However, a higher number of working hours was shown to decrease physical activity in the inpatient care sector [48]. In addition, according to a cross-sectional study from Thailand, nurses in training already seem to show little to moderate physical activity [51]. Another study showed that fatigue due to long working hours was a strong reason for Australian nurses not to become physically active [52]. Alternating night shifts have been shown to reduce physical activity during leisure time, both in inpatient care and among midwives [57]. Existing stressful working conditions, such as a demanding working environment or shift work, are also possible reasons for low physical activity and the resulting high number of overweight inpatient nursing staff [60]. Compared with other hospital professions, inpatient nurses show the lowest level of physical activity [41]. Training in geriatric and nursing care in Germany is already characterised by low physical activity (<2 h/week) during leisure time [55].

#### 1.2.3. Smoking Behaviour

Nurses, in contrast to other hospital occupational groups, showed lower levels of smoking behaviour [53]. However, looking at the employees of inpatient care, it becomes clear that high tobacco consumption can be attributed to job-related stress: Weekly working hours that are perceived as negative (e.g., location, length) are associated with increased smoking behaviour as well as with a lack of sleep [42,43]. Even in training, caregivers already show regular nicotine consumption [44]. An examination of New Zealand’s smoking prevalence among doctors (*n* = 12,684) and nurses (*n* = 39,126) revealed that nurses smoked more than doctors (17% compared with 2% in 2013) [54]. A systematic review and meta-analysis by Nilan et al. [61] underlines that there could be an association between a healthcare occupation and smoking prevalence: compared with other healthcare occupational groups, nurses showed significantly higher tobacco use than doctors, dentists, and pharmacy employees (*p*-value < 0.01) [61]. The microcensus is the official representative statistic on the population and the labour market in Germany. According to the microcensus (data basis: 2017), 3,082,000 medical health professions are regular smokers; of them, about 36% are found in healthcare and nursing (*n* = 1,118,000) alone [62]. A high smoking prevalence among caregivers working in the German inpatient care setting is therefore assumed, whereas there are no studies describing the smoking behaviour of German outpatient caregivers (see [26]).

#### 1.2.4. Breaks and Regeneration

The scientific literature increasingly refers to the insufficient implementation of breaks in practice among nurses. This is often due to the delay of breaks or interruptions during the break time [63]. On the basis of data from the employment survey conducted by the Federal Institute for Vocational Education and Training (BIBB) in cooperation with the Federal Institute for Occupational Safety and Health and Occupational Medicine (BAuA)—BIBB/BAuA survey of employed persons—there is an increase in the number of nurses (including inpatient and outpatient nurses, *n* = 746), and there is an increase in the loss of breaks due to overtime, time pressure, and shift work. Furthermore, disturbances/interruptions and excessive demands due to too high a workload are reported [64]. Outpatient nurses work in a very special setting, as they have to be constantly on the move in order to reach their patients and to be able to carry out their nursing work in the patients’ own homes [9]. Therefore, the extent to which German nurses can recover during working hours in the outpatient setting should be evaluated [26]. The conditions of work organisation inherent in the activity pose a particular challenge. The time and performance pressures to which caregivers are exposed make it difficult to comply with legally required breaks. Turning down a break opportunity can be interpreted as a “time-saving coping behaviour” [65]. Furthermore, understaffing has also been shown to be a facilitating break failure factor in nursing [66]. It is also striking that among inpatient nursing staff, smokers seem to be more active in taking their breaks compared with non-smokers: Tobacco addiction was satisfied by claiming regular breaks [45]. Shift work as well as work demands have already proven to be detrimental in inpatient care with regard to sleep quality and the implementation of health-promoting behaviours (physical activity, sufficient regeneration) [42,46]. A short recovery sleep during working hours was associated with better recovery after the shift for inpatient nurses [47]. However, to date, little research has been done on the break and regeneration behaviour of German caregivers [26]. Sleep quality was also negatively influenced by violence at the workplace among Chinese hospital nurses and led to higher perceived stress [50]. In this regard sexual harassment is also a known serious source of stress in the outpatient care setting [9,67].

#### 1.2.5. Stressors, Stress Perception in Care and Potential Influence on Health Behaviour

With regard to the health of employees, care professions are characterised, among other things, by the “combination of high mental and physical demands” [68]. An integrative review by Broetje et al. [69] analysed 14 reviews regarding key job demands and key resources in nursing staff (hospitals and nursing homes) in reference to the job demands–resources model (JD-R Model) by Bakker and Demerouti [70]. Main job demands were an overload of work, missing rewards in terms of payment/benefits, and a high interference of work with private life. Main resources were the social support of supervisors, a professional management built on trust and fair leadership as well as respect, autonomy, and interpersonal relationships with colleagues and patients, and lastly, occupational resources (e.g., work organization and equipment) [69]. Research by Bakker et al. [71] focussing on home care nurses (*n* = 3092) mentions workload, time pressure, working hard, physical demands, emotional demands, and problems with planning the work day as specific job demands and stressors, while additionally having to confront sexual harassment and patient harassment. Resources were autonomy, social support, coaching by a superior, professional development, performance feedback, and financial rewards [71]. In the international context, there are various studies that examined risk factors, stressors, the stress experience, and their consequences on care workers as well as their interrelationships. For example, in a cross-sectional survey of psychiatric nurses in Ireland, the lack of trained staff and a high workload were found to be stressors. High workload was also found to be a significant predictor of emotional exhaustion (*p* < 0.05) [72,73]. Furthermore, research findings on nurses show that high work demands combined with a lack of social support and little opportunity for decision-making lead to depressive symptoms and emotional pressure and may even lead to burnout [74,75]. In a large-scale Taiwanese cross-sectional study, inpatient nurses (*n* = 31,639) showed unhealthier behaviours in terms of balanced diet and physical activity compared with physicians (*n* = 4202), pharmacists (*n* = 2315), and other hospital staff (*n* = 21,240) [41]. However, there are only a few recent studies on the triggers and stress experience and their long-term consequences on German care workers [55,70,76,77,78,79,80]. Nevertheless, research results from Germany highlight time pressure and work overload as key stressors in the care sector [64,68]. In the BAuA’s survey of employed persons, almost a third of the participating care workers stated that they often work at the limits of their capacity to perform [64]. Social support (at work and in private), appreciation, and communication have proven to be important resources in the care setting that can counteract the individual experience of stress and potential strain reactions, such as depressive symptoms [74,75,79,81,82]. Resilience and self-care training could enable caregivers to better cope with work demands [83] and to show enhanced health behaviours [16].

Despite the differences between inpatient and outpatient care settings, the generalizability of knowledge might be possible to a certain extent [26,84], as there seem to be similar demands and resources in outpatient care (see [85]). Although first results about straining working conditions of caregivers (primary of the hospital nursing staff) exist (see [86]), there is hardly anything known about German outpatient caregivers’ health behaviour [26]. However, with respect to the high sickness rate for German outpatient caregivers, there is great prevention potential in establishing healthier working conditions in outpatient care. Outpatient caregivers work in a special setting, as they are constantly on the move and work at the patient’s home, as opposed to inpatient care [9]. Therefore, because of these special working conditions in outpatient care, the choice of health-promoting behavioural patterns as well as the conduct of health-promoting behavioural patterns are assumed to be more complicated [16,87].

As a consequence of the different workplace settings and the associated framework conditions which could influence inpatient and outpatient caregivers’ health behaviour, study results from inpatient care are only generalizable to a limited extent [26]. Moreover, there is little known about outpatient caregivers’ health behaviour overall. However, according to a systematic review, it is assumed that their personal health behaviour might have an impact on their health-promoting practices [88]. Ultimately, there are no studies analysing specifically German outpatient caregivers’ health behaviour as of yet, studies that simultaneously focus on eating behaviour, physical activity, smoking behaviour, and rest and regeneration behaviour [26] and that focus on the factors that might have an inhibiting or promoting effect in this context (see Table 1).

Thus, the aim of this study was to investigate the health behaviour of outpatient caregivers. We proposed the following research questions:

1. In what way do outpatient caregivers apply their health behaviour (nutrition, exercise, smoking, regeneration)?
2. Which personal health-promoting behavioural patterns are exhibited by German outpatient caregivers?
3. What factors do they experience that influence their health behaviour (e.g., stress, coping, support)?

## 2. Materials and Methods

### 2.1. Subjects

Participants were outpatient caregivers who had been employed by outpatient care services in Northern Germany for at least six months. Outpatient caregivers were eligible if they were at least 18 years old, had been providing care at the patient’s home and thus had to travel from the company to the patient. The qualifications of the participants were not determined in advance and did not play a role; only the activity in outpatient care was relevant. Outpatient caregivers had to work at least part-time. Informed consent was obtained in advance, and participants agreed in writing to participate in the interview. This study was supported by the Institution for Statutory Accident Insurance and Prevention in the Health and Welfare Services (BGW), a non-profit organization based in Hamburg, Germany, that is part of the national social security system.

### 2.2. Study Design

The present study followed a qualitative research approach, as the qualitative research approach was considered to be the most appropriate for obtaining initial findings in a new field of research [89]. Outpatient caregivers’ opinions and experiences are at the centre of this study [90]. “Subjective truth” and the reconstruction of personal experience were strived for [91]. Prior knowledge through literature research and observation (*n* = 6 participatory observations of different working shifts of outpatient caregivers) built the basis of the questions that were asked in the interview [92]. Interviews were carried out in a combination of face-to-face interviews (*n* = 7) and telephone interviews (*n* = 8). All steps—from conducting interviews to transcribing interviews and analysing data were carried out by the first author, a female health scientist (NM, M.A. Public Health) who works in the field of “occupational health psychology”.

### 2.3. Participant Selection and Interview Conduct

In total, we conducted 15 problem-centred interviews (as recommended by Witzel [93]) with outpatient caregivers from Northern Germany, following a deductive–inductive procedure [94]. The problem-centred interview method was chosen as it is used to record specific behaviour, experiences, reasons, evaluations, and subjective opinions in a dialogue and aims for a process that results in a common understanding between the interviewer and the interviewee [93,95]. A total of 15 interviews were chosen because the repetition of experiences and attitudes in the interviews revealed a theoretical saturation of this study [91,96]. Eight of the interviews needed to be carried out by telephone due to accessibility issues of outpatient caregivers. Two interviews were conducted in January 2020, six were conducted in February 2020, and the remaining interviews were performed as telephone interviews in March and April 2020. Study participation was voluntary. Prior to the interviews, interviewees were asked to sign a declaration of informed consent regarding the performance and recording of the interview. All participants were in a position to understand and consent to the study requirements, and all provided written informed consent. A purposeful sampling was applied. Individuals who were working as an outpatient caregiver for at least six months in the same care service (in small- and medium-sized enterprises in Hamburg) and who were fluent in the German language were eligible and were recruited. No specific occupation in outpatient care was presupposed. Outpatient care services were contacted via invitation emails and telephone calls by the interviewer (NM) herself. All interviews were tape recorded. Interview length was from 23 up to about 65 min. Participants were told that they were able to terminate the interviews at any time. No non-participants were present during the interviews. No repeat interviews were carried out. Field notes were made immediately after each interview.

### 2.4. Interview Guideline

Within the general framework of the empirical and theoretical background, a semi-structured interview guide was designed. Interview questions were collected, reviewed, and sorted. Afterwards, questions were subsumed in categories [91,97]. Following Misoch [98], the interview guideline was divided in four phases (information phase, warm-up phase, main phase, end of the interview). An extract of the interview guideline is shown in Table 2. A pre-test interview was performed before the actual first interview in order to receive feedback from research colleagues and to improve the interview guideline where applicable. The complete interview guideline can be found in Appendix A in Table A1.

### 2.5. Data Analysis

All audio recordings of the interviews were transcribed verbatim following Kuckartz [92]. Subsequently all transcripts were anonymized and analysed in a deductive–inductive process according to the qualitative content analysis of Mayring [99]. The code system can be found in Appendix B in Table A2. We used MAXQDA 2020 (VERBI Software, 2019) (VERBI GmbH, Berlin, Germany) for data analysis [100]. In an iterative process, the main researcher identified and refined codes, categories, and sub-categories. Coding was reviewed reciprocally for accuracy and was carefully debated with the head of the research group until consensus in terms of the final coding system was attained. Another separate document served as the final coding system. The material was then further reduced and compressed by the main researcher (NM). During the process of analysis, reflexivity and transparency relating to the potential influence of the researchers’ objectives and prejudices on the results as well as interpretations were constantly emboldened. Transcripts and results were not returned to the interviewees, although they were allowed to request them at any time. All quotes used in this report were translated from German to English. The COREQ checklist (consolidated criteria for reporting qualitative research) was used to ensure the quality of reporting on the methodology of this qualitative study [101].

## 3. Results

### 3.1. Sample Characteristics

As shown in Table 3, interviewees were between 21 and 67 years old. Of those interviewed, 15 were outpatient caregivers from Hamburg, Germany, 12 were female, and 11 worked full-time; their work experience ranged from 1.5 up to 31 years at the time of the survey. Thus, all of the participants had been working in the outpatient care sector for at least six months, as was required to be eligible. Most of the 15 interviewees were qualified as geriatric nurses or caregivers. Interviewees #10 and #12 had other qualifications which were not relevant to the profession (storekeeper and interior decorator) since they slipped into the care profession while working as temporary staff.

The work activities of the outpatient caregivers mainly involved the care of patients. Putting on compression stockings, positioning patients, and showering those in need of care were mentioned particularly frequently. In addition, the administration of medication, wound care, and changing protective pants for bedridden patients were also reported. The additional conduct of staff appraisals was stated by one interviewee.

### 3.2. Health Behaviour of Outpatient Caregivers

From the interviews, the following seven main categories relating to health behaviour were identified: eating behaviour, drinking behaviour, physical activity, smoking behaviour, break behaviour during work, regeneration after work, and personal health-promoting behaviour.

#### 3.2.1. Eating Behaviour

Eating behaviour in general varied among interviewed outpatient caregivers. Responses were given about their own perception of eating habits, times and places of food intake, food choices, snacks, and the wish of eating better while looking at the impact their job has on their eating behaviour. A lot of them described their nutritional habits as good, as they reported cooking fresh food or eating a balanced diet, among other things. Additionally, breakfast before work was emphasised by a few. In contrast, however, a large proportion of the outpatient caregivers described their diet as in need of improvement. Reasons for this varied, with eating in front of the television and the irregularity of meal intake being named.

*“I would say rather balanced. Well, yes, I’m not like that, I don’t know, I somehow just pay attention to eating healthy. Really, actually balanced. Well, I, yes, we cook fresh, I’ll put it that way, we’re not fast-food eaters here, but even that happens from time to time, so therefore it’s actually quite balanced.” (Interviewee #5)*

The majority of respondents ate during their shift or in between on their tour. Rarely, specific times were described, resulting for example from the break or a regular joint breakfast with the team.

*“In between, when I’m sitting in the car, you could certainly eat something, yes.” (Interviewee #11)*

Most interviewed people mentioned their car as the most frequent place of food intake, especially when they perceived work as stressful or felt time pressure. Some reported using the (existing) break room or the office in the company for food supply. Others were forced to eat in the parking lot, on their bicycles, or at the bakery. The way home on the bus or the nearby park in good weather conditions were also mentioned.

*“During the ride, when I/[I: So in the car?] Yes, exactly. When I’ve somehow finished with a patient or when I see that I’m well on time, then I can stop briefly in the parking lot when I’m with the next patient and then have a quick breakfast. But actually it’s always during the ride, yes.” (Interviewee #5)*

Food choices varied among the respondents. In part, a (ready-made) salad was consumed in order to eat healthily or to reduce weight. Some outpatient caregivers did resort to vegetables, fruit (especially banana and apple), and smoothies as well as home cooking (fish, meat, pasta, rice), or they paid attention to having a sufficient protein intake (shakes or bars). Most of them, however, ate ready-made meals to heat up (on the go during or after work). Pizza, burgers, chips, and especially baked goods (bread, rolls, croissants) were increasingly on the menu. In addition, many reported regular consumption of chocolate, sweets, cakes, or crisps. In contrast, fewer opted for nuts to chew on during work.

*“Well, in the morning I have something sweet from the bakery or something, right? Maybe a piece of fruit, which would actually be healthy, but rather rarely.” (Interviewee #2)*

The time pressure at work favoured the choice of snacks in between. However, some respondents said that they snacked on fruit (e.g., banana, apple, grapes) or vegetables (such as carrots) while at work. The majority, however, liked to snack on sweets in between meals, e.g., in the form of cakes or chocolate/chocolate bars. Nuts or muesli bars were snacked on less frequently.

*“Yes, if there are sweets lying there, they are mine. They will be gone to 100%. So I grab them, because anything that’s edible and I can grab it while I’m doing something, it’s gone. The first thing I did this morning was grab a cake, because it was sweet.” (Interviewee # 6)*

Moreover, outpatient caregivers reported a general impact of their job activity on their eating behaviour. The sudden irregularity of individual meal taking was most frequently emphasised. For example, in this context, the outpatient caregivers stated that they could not eat in peace during work. According to the respondents, a more balanced diet was more possible on days off. Overall, the consumption of sweets and fast food had increased.

*“Changed, changed. Yes, this irregular eating, right? Before, you had a schedule, so you could divide it up a bit. Breakfast in the morning, then lunch at noon. Evening meal, not necessarily, but so that you paid attention to it. And so, yes, I would say it changed. Because I can’t do it in the morning either, or I don’t have breakfast before work, a black coffee is enough for me and then, yes, depending.” (Interviewee #4)*

Ultimately, the majority of outpatient caregivers expressed the desire for a healthier diet. However, in this context, they faced some challenges in being able to implement a healthier diet. On the one hand, the lack of time in everyday life was put in the foreground, so that organisation and the motivation to cook healthy food were impaired. In turn, time constraints at work, which could also sometimes lead to missed breaks, meant that they did not have time to pick up food and then eat it. The high responsibility towards patients was mentioned as one of the reasons. Thus, something was usually eaten in the car; the spatial possibilities on tour are limited. For some, the early shift was also too early to eat something beforehand.

*“Yeah, sure, I mean healthier is always possible. Why not.” (Interviewee #12)*

*“We don’t take a break, and when we do, I always have to make something or take a bite somewhere in between. So it’s really always just one bite and then it continues. There is no regularity.” (Interviewee #11)*

In addition, stress and hectic pace encouraged the choice of unhealthier foods among the respondents. Therefore, sweets would be consumed more frequently by the outpatient caregivers when there was less time. However, loss of appetite was also mentioned.

*“Yes, when I’m stressed at work, I feel like eating sweets.” (Interviewee #7)*

#### 3.2.2. Drinking Behaviour

Interviewed outpatient caregivers talked about their drinking behaviour regarding beverage choice, how much they drank, and when it was possible for them to drink. Influence and challenges through work were also mentioned. Other than water, the beverage choice of the surveyed outpatient caregivers was varied. Most of them stated that they drank coffee every day, which was either sweetened (sugar or sweeteners), black, or with milk. Many of the respondents drank tea every day, whether with sugar, honey, or lemon. Juices, soft drinks, or wine were drunk less frequently. The amount of fluid intake varied. While some felt they were drinking enough (1.5–3 L/day), some other outpatient caregivers described their daily drinking amount as insufficient (0.5–1 L/day).

*“I drink my coffee in the morning when I start and I take a bottle, because we also have free water here, free drinks, and then I take a bottle of water with me and just drive off.” (Interviewee #8)*

*“Well, I always have to force myself to drink a lot, so I always try to get to a litre during the shift.” (Interviewee #5)*

Most of the respondents also stated that drinking between activities during their work was generally always possible. Several times the car or the office were mentioned as places where they could drink.

*“In the car and accordingly also when I say there is no eating in my car, but I can at least always have a sip in the meantime before I start, so to speak, to take another two, two sips, to drive to the next customer.” (Interviewee #9)*

The outpatient caregivers highlighted some of the challenges regarding daily hydration. In addition, they mentioned that more can be drunk on days off compared to workdays. A large number of the respondents highlighted that during outpatient care work, the possibilities to visit a toilet are very limited. Therefore, less drinking was done in advance. Sometimes there are no drinks available, and there are no refilling possibilities. The experience of stress and time pressure during work were mentioned as reasons that strongly favour forgetting to drink.

*“Well, sure, I take something with me. That’s quite clear, it’s just always the same with going to the toilet, because of course you can’t go everywhere. So I already have my route that I take, where you know where you can go and where not. And of course you always have to look a bit. I always plan to drink a 0.7 bottle of water, but that often doesn’t work.” (Interviewee #1)*

*“So two litres a day in any case. But that’s also a problem because of the work, because we have to go to the toilet a lot and that’s also difficult. That is the disadvantage of outpatient care. And if I don’t drink enough, I have a headache, that’s also a disadvantage then, that’s why/the distances also increase sometimes, often I come to the office. I don’t like to go out somewhere. Well then, that’s just the way it is. A bit stressful over time.” (Interviewee #7)*

#### 3.2.3. Physical Activity

The interviewed outpatient caregivers reported on their physical activity during and after work or in their free time, mentioning also potential barriers regarding their physical activity. According to their own statements, most outpatient caregivers reported being very active during work due to their job itself. Some of them travelled to their patients by bicycle as opposed to driving a car, so they were basically additionally active. Furthermore, it should be emphasised that many outpatient caregivers take care of patients who live on upper floors, and not all houses have lifts. If lifts are available, the preference varies; the stairs are taken by most respondents up to the fifth floor, for example. Furthermore, one interviewee emphasised that she takes 7000 steps in an early shift (measured with her own pedometer).

*“So that’s/you’re from one patient to the next, stairs up, stairs down, sometimes without lifts. There is a lot of movement. Getting patients out of bed, putting on compression stockings, etcetera pp, that’s already/I measured once, I don’t know, I think it was 7000 steps in the early shift.” (Interviewee #11)*

Their physical activity in their free time varied. Only a few outpatient caregivers reported participating in regular sports, such as jogging or sports courses. More of them liked to go cycling, swimming, or hiking in their free time. Home training or going to the gym was less common. Although some of them did yoga or Pilates, a large part of the respondents also stated that they were physically active irregularly or not at all.

*“I go to the gym twice a week. That’s all I can do.” (Interviewee #2)*

Most of the outpatient caregivers interviewed expressed a desire to become more physically active in general. In this context, however, many respondents reported that since they started working in outpatient care, their physical activity in their free time had been considerably reduced. In the course of this, they mentioned some challenges from their work activities. Most outpatient caregivers do not have the energy to do physical activity after work. In contrast, many of them lack the necessary time after or before work, which is limited by shift work. Others, on the other hand, said they were too lazy and did not feel like it. Private reasons (e.g., financial aspect) as well as stress or pain also played a role for those respondents whose physical activity was too low.

*“Yes, I think that being a/really physically very demanding/at least for me I can say that I don’t necessarily feel like it and I’m just very tired when I’m at home. And then going to the gym somehow, I did that for a while, but it’s not really what fulfils me. I’m simply exhausted, no, I’m happy when I have my peace and quiet and can sit, at least for a while, I mean, you don’t sit at home straight away, but in that respect you do. It’s just not an office job.” (Interviewee #10)*

#### 3.2.4. Smoking Behaviour

Many of the outpatient caregivers interviewed were smokers and used cigarettes regularly. One respondent even replaced cigarettes with nicotine spray before the interview. Many outpatient caregivers stated that the need to smoke was especially increased when their individual stress level at work was higher. For some of them, time pressure also played a favourable role. Many of them did not feel like using tobacco at certain times but said that they would do so every few hours in between. Many mentioned that they would smoke during their break. It was striking that one respondent said that they smoked only at work and barely when they were at home.

*“When I’m stressed, I smoke a cigarette.” (Interviewee #14)*

In this context, the influence of the work activity of outpatient care on the respective smokers became clear: it was mentioned several times that constantly being on the move at work favours smoking. Furthermore, it was emphasised that individually perceived work stress and time pressure led to increased smoking. One interviewee had stopped smoking but started smoking again after starting work as an outpatient caregiver.

*“Well, I don’t smoke at home as a general rule, and if I do, it’s only at work. (…) I even started again at work.” (Interviewee #2)*

Nevertheless, some interviewed outpatient caregivers reported that they had the intention to quit smoking. However, they also reported that work activities (e.g., experienced work stress) present counteracting challenges.

*“((laughs)) Yes, if it were that easy, then, then I would like to stop.” (Interviewee #10)*

#### 3.2.5. Break Behaviour during Work

Breaks during working hours were perceived as recreation by the interviewed outpatient caregivers. The frequency of the breaks depends on the respective shifts. Most of the interviewees reported that they are entitled to a break of 1 × 30 min during a full shift. It was often emphasised that shorter breaks were taken more often instead of a long break, since they can get more rest in between or because it is hardly possible due to the tour planning. The part-time workers stated that they did not have a break. Furthermore, one interviewee who used to work in inpatient care emphasised that breaks were always possible during inpatient care work, in contrast to outpatient care work.

*“Well, I have a set half hour a day. And then, as I said, always in between, as it suits me. If, let’s say, I finished a patient a bit quicker or the next patient is half an hour later or something, then I try to sit down again for a short while and have a drink. Yes, always in between, when it suits the time and during my break.” (Interviewee #13)*

The outpatient caregivers’ break arrangements varied. The majority of them spent their break in the break room (if available) alone or with colleagues who also took their break. Others could take their breaks in the office, where they could sit down and have something to eat or drink. A few respondents reported taking their break on a bicycle to the next patient. The outpatient caregivers who smoked associated their break with tobacco use.

*“I sit, I smoke, I drink coffee. I don’t answer the phone. I chat ((laughs)).” (Interviewee #6)*

Lastly, the majority of the interviewed outpatient caregivers mentioned break failure. This inability or reluctance to take a break was evident in almost all respondents. Reasons for not taking a break were mostly, e.g., lack of time, tight tour planning, experiencing stress, and unforeseen extra work.

*“To be honest, I can’t take this break at all like this, because we have planned a travel time of five minutes between two patients and you can’t even make it from one patient to the next in five minutes.” (Interviewee #4)*

#### 3.2.6. Regeneration after Work

Regeneration after work was described by the respondents in terms of available recreational opportunities and individual sleep. Recreational opportunities varied according to individual preferences. For many of them, physical activity or sporting activities was a strategy for recovery. However, most of the interviewees also reported that reading a book, going out for a meal or a drink, and simply taking a nap or doing nothing were perceived as ways to relax. However, some also mentioned spending time with family or pets or watching television in this context. Less frequently, mental hygiene, photography, or gardening were mentioned.

*“I have this routine that I come home. I now have a cosy sitting area in my kitchen where I first relax, have another drink. Maybe I have another short chat with my daughter or prepare dinner or whatever. Or simply come home and turn on the TV and say, I’m going to let myself be entertained for a while.” (Interviewee #9)*

The respondents described their nightly regeneration through sleep differently. Most of them regularly slept less than 6 h. However, almost all of them mentioned that they slept more on days off. In this context, the outpatient caregivers described some negative influences on their sleep quality that were the result of their work activities. Sad images from the work activity, e.g., when patients are very ill, which causes pity, were mentioned as a cause. Other reasons were perceived work stress in general. Shift changes and especially early duty were mentioned as the most common factors affecting personal sleep quality.

*“Well, it has the effect that I’m exhilarated, that I can’t sleep because there are images running through my head. (...) There are also things that get to you. Really bad things. We also often get short admissions, people who are discharged from hospital and then come home to die or have really bad things that heal, but still/it is also rare, but there are also things that already/so rarely/actually almost always have someone where one now also has pity, right? So now beyond the professional then.” (Interviewee #1)*

*“For example, when I’m on early duty/I can’t fall asleep so quickly. That’s why I always go to bed late. And then I get up early, which is different from when I’m on late duty, because when I’m on late duty I’m home at about 12 o’clock at night. Then I go straight to sleep when I get home. And then I sleep until nine or ten. I find it very different from early duty, somehow.” (Interviewee #14)*

#### 3.2.7. Personal Health-Promoting Behaviour

The surveyed outpatient caregivers reported that their personal health-promoting behaviours differed based on whether or not they were working. During work, most respondents implemented back-friendly work to protect their own health. In addition, many regularly disinfected and/or washed their hands. Wearing personal protective equipment (PPE), such as face masks, gloves, and gowns when interacting directly with patients, was also mentioned in this context. Voluntary cycling during work to keep moving and staying calm as well as driving carefully were also reported. Only one interviewee mentioned taking breaks.

*“(…) Even when putting on compression stockings, I try to maintain the correct posture and not bend over too much or slouch, because it also kind of affects my back. (…), sometimes you have to go through it even if you already have severe pain in your back, working in a bent position or on your knees, so to speak, because the home environment and conditions of the patients do not allow it. (…) you have to make sure that you work healthily and gently for yourself.” (Interviewee #4)*

Finally, personal health-promoting behaviours related to private life were described in terms of sport and exercise, massages (if financially possible), and time with family and friends. Most, however, emphasised healthy eating in this context.

*“I try to eat healthy as much as possible. Yes. Maybe I should go cycling or something more often in my private life. So I don’t really do that much.” (Interviewee #10)*

### 3.3. Factors Which Could Influence Outpatient Caregivers’ Health Behaviour

Almost all respondents described that they perceive stress at work on a daily basis. In this context, the outpatient caregivers reported several demands they faced during their work, which are explained in the following.

#### 3.3.1. Stressors in Outpatient Care That Hinder the Implementation of Health-Promoting Behaviours

All interviewed outpatient caregivers agreed on the issue of time constraints. According to the respondents, the tours are planned too tightly. Staying longer with patients was unavoidable. Overall, the time allowed by the health insurance companies was too unrealistic and not feasible in practice. In addition, traffic-related challenges, such as traffic jams or road works, led to delays and thus caused time constraints. In this context, many respondents emphasised the constant search for parking spaces which caused time delays. Furthermore, some respondents felt that the physical exertion during their work was stressful. Many also mentioned not knowing whether or not their patients’ health had deteriorated in the meantime or even whether a patient had died. Many of the interviewees also complained about a lack of support in the team or existing hierarchies between superiors and employees in the care service. Unclear responsibilities/competences, no help from colleagues in the immediate vicinity, and on-call duty were also named. One interviewee reported on the demands resulting from an empty tank of a shared car. Some outpatient caregivers also found sick leave from colleagues to be stressful as it resulted in a higher workload.

*“Yes, at most it would be when you have unexpected things like traffic jams and traffic and not being able to find a parking space. Those are the kind of things that just stress you out and get on your nerves, yes. Or lifts are broken and then you have to go to the 16th floor and [I: 16th really?] Mhm, that also happens sometimes, yes. Then it’s ((laughs)) hard.” (Interviewee #5)*

#### 3.3.2. Individual Coping Strategies as a Supportive Factor for Realizing Health-Promoting Behaviours

Coping strategies were described on the one hand for work and on the other hand in the private lives of the outpatient caregivers. During their shift, most respondents told us that they would try to calm themselves down. In this setting, they would take deep breaths to calm down. A very large proportion of respondents would go out for a cigarette to compensate for perceived stress. However, a few also reported that they would accept the situation or avoid the stressful experience. Some outpatient caregivers also mentioned snacking on sweets as a coping strategy during work. A few respondents reported that they curse while driving and swear at other road users.

*“Yes, when I was stressed, I smoked more.” (Interviewee #1)*

During their free time, the outpatient caregivers partly resorted to other coping strategies. Communication with family, partners, and friends was mentioned most frequently. Going for a walk, doing sports, or simply keeping moving were also mentioned by the respondents. In this context, those owning a dog used the daily walk with their dogs as a coping strategy. Some of them talked about relaxation techniques, such as yoga or progressive muscle relaxation or about sleeping the stress off. Listening to music, reading, or doing handicrafts as well as watching TV were also described as strategies for coping with everyday stress.

*“Staying at home, watching a bit of TV. Or, when I’m really stressed, I call a friend. And then everything is gone.” (Interviewee #7)*

#### 3.3.3. External Support in Regard to Advice or Assistance: Private and Workplace Aid

Existing support when it comes to advice or assistance was reported on a private level and on a workplace level by the outpatient caregivers. Regarding support by employer or institution, the interviewed outpatient caregivers experienced internal work-related support mainly from their team, either through colleagues or their management. The respondents described external support in terms of labour law, the Federal Association of Private Providers of Social Services, and the VERDI trade union. However, some outpatient caregivers also mentioned that support from employers was completely lacking because the boss does not want to be disturbed.

*“They’ll take over a patient if I’m too stressed. They can do that. Yes, once a patient is off the schedule, so to speak, then everything is fine again.” (Interviewee #14)*

Moreover, outpatient caregivers received private support mainly from their families. Family members and partners but also children were mentioned by the respondents. In this context, most outpatient caregivers referred to private support from friends who, for example, worked in care as well or simply had an open ear. A few emphasised that they would not receive any private support. In general, however, they reported that they did not like to mix their private lives with matters from their jobs.

*“Yes, I have a friend who is also a nurse. She understands this quite well. But she is an inpatient nurse. With the—of course—family, but you always try to do that a bit/work is work and private is private.” (Interviewee #2)*

**Figure 1:**
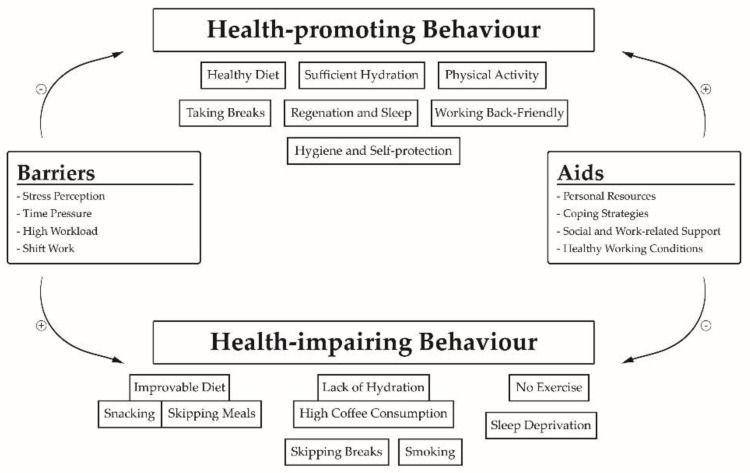
Outpatient caregivers’ health behaviour, as well as factors influencing health behaviour patterns.

A summary of the results regarding the health behaviour of the interviewed outpatient caregivers and their described barriers and aids regarding the implementation in practice is shown in Figure 1.

## 4. Discussion

This is the first study to apply qualitative methods to a comprehensive examination of the health behaviour and health behaviour-influencing factors of outpatient caregivers in Germany. Important insights into the working conditions and the associated health behavioural patterns were gained. Outpatient caregivers have a special work setting with certain stressors which could have a negative impact on adopting health-promoting behaviour. Interviewed outpatient caregivers showed different diets as well as different drinking patterns. Even though there was a desire for a healthier diet or increased fluid intake, the respondents faced many challenges. While the work activity itself provided sufficient physical activity during the shift, in most cases it prevented physical activity during leisure time. Many outpatient caregivers smoked when they were stressed or when taking a break. However, the interviewees’ statements emphasized that the opportunities for breaks during work were sometimes limited. The regeneration behaviour of the interviewed outpatient caregivers consisted of their sleeping behaviour and also restorative activities. In addition to the differentiated health behaviours suggested above, we were also able to examine health-promoting behaviours, such as the use of PPE or disinfectants, from the perspective of outpatient caregivers. Most of the interviewees talked about a high perception of stress during their daily work life due to specific job-related demands, such as time pressure and a constant high workload. To cope with the stress at work, a lot of outpatient caregivers mentioned smoking or snacking. Supportive factors were communication in the team or private support from family members or friends.

### 4.1. Health Behaviour of Outpatient Caregivers

In the course of their nutrition, the interviewed outpatient caregivers showed different behaviours. It is generally assumed that human beings prefer foods that provide a high energy intake (fats, carbohydrates) at minimal cost (financial and time) and at the same time require little effort (procurement, preparation and cleaning) [102]. In this study, the outpatient caregivers described their diets as being in need of improvement. Many of them ate something quickly in between meals or in the car because they felt they had no other choice. They mostly ate ready meals, fast food, baked goods, or sweets. Study results from Germany, which refer to geriatric care, show that compared with inpatient nurses or educators, nutritional behaviour among geriatric nurses is worth improving [55]. A qualitative study by Power et al. [103] among inpatient nurses also supported our finding that shift work and stress in particular made it difficult to implement a healthy diet. Other study results from inpatient care settings also show associations between shift work and a nutritional pattern in need of improvement [38,39,40,48,52,104,105]. These research results are not related to the outpatient care setting specifically, but nevertheless, generalizability—albeit limited—is possible (see [26]). The respondents in our study also showed that they tend to have comparatively more negative dietary behaviour when they are stressed; choosing sweet foods is increased when stress perception is heightened. This is also reflected in other studies from inpatient care. Increased stress perception from work has been associated with increased calorie intake, (e.g., [37,38]), or elsewhere has even contributed to the development of an eating disorder [106]. Moreover, older caregivers looking after family members show signs of malnutrition and obesity that is associated with depressive symptoms [107]. Snack intake among the outpatient caregivers we surveyed was very high. Shift work might increase snacking behaviour [39,108], and further study results show that canteens and break rooms could encourage caregivers to eat regular meals (see [40]. However, these are often lacking in outpatient care, especially given that the mobile nature of the work might lead to the expectation that workers are more likely to reach for snacks between meals [26]. Irregular meal intake could also indicate poor sleep quality, according to recent study results on nursing students [58].

The drinking behaviour of the outpatient caregivers was similarly reported by the interviewees. Our study results highlight that without exception, all outpatient caregivers surveyed consumed coffee in large quantities on a daily basis, especially during their work shift. Considering research results, inpatient nurses and midwives show increased coffee consumption, especially when working shifts [39,56,109]. Caffeine is known to reduce symptoms of fatigue [110]. Constant pressure to perform, as is already known in care, could thus favour the desire to consume coffee (see [32]). Nevertheless, many respondents stated that they did not drink enough during the day in general. The main reason in this context was the lack of opportunity to visit a restroom. This challenge was also highlighted in a recent study by Pierce et al. [111] on voiding delay, fluid restriction, urinary symptoms, and work productivity among female inpatient nurses and midwives. Holding the bladder because of limited toilet facilities inevitably led to lower fluid intake and ultimately resulted in a loss of work productivity [111]. The lack of toilets can also cause outpatient caregivers to neglect adequate hydration due to their location-unspecific working environment [26].

In the present study, the outpatient caregivers interviewed described what they considered to be sufficient physical activity during their work activities, for example, by constantly climbing the stairs to the patients or cycling the tour. However, during their free time, only a few respondents reported physical activity on a regular basis. This is also reflected in other study results: Compared with inpatient nurses and educators, geriatric nurses showed the lowest physical activity according to Wirth et al. [55], which was also seen among family caregivers [112]. Moreover, it appears from inpatient nursing that inpatient nurses are less physically active during their leisure time compared with other hospital occupational groups, (e.g., [41,48,52,57,104]), which is also already seen among nurses in training, (e.g., [44,51,58]). A few respondents in our study were physically active on a regular basis, which also seems to be the case less frequently in other research results from inpatient care [53,60]. In this context, most of our respondents emphasised encountering challenges such as fatigue due to work overload or the changing shifts at work. Fatigue and shift work are also factors that impede physical activity during leisure time for inpatient nurses or midwives [48,52,57,103,104], as well as stress perception [103].

Most of the outpatient caregivers interviewed were in agreement with regard to behaviour that impairs health. Many of our interviewed outpatient caregivers were smokers. There are first indications that the adults of Germany’ population are smoking less overall: 26.6% of women and 35.0% of men have stopped smoking based on the data of the GEDA 2014/2015-EHIS study (“Current Health in Germany”) [27]. According to an exploratory focus group discussion with experts from the Hamburg care sector, smoking behaviour also seems to have improved [113]. However, smoking is highly prevalent as a health-impairing behaviour in the care sector compared with other healthcare workers [42,43,44,54,55,61]. Geriatric nurses from Germany also show the highest smoking rate compared with in-patient nurses and educators [55]. Many of our respondents combined smoking with taking a short break, which has also appeared in other study results among nurses [45]. Perceived work stress and time pressure were factors that especially favoured smoking among outpatient caregivers. In this context, high tobacco consumption is also known in inpatient care, which is mostly associated with job-related stress perception, long working hours, shift work, and duties towards family members [42,43,104].

The respondents described their break behaviour or rather the use of breaks as difficult. Regarding rest during working hours, the majority of our respondents reported a complete absence of breaks even though the German Working Hours Act (ArbZG) stipulates minimum breaks [114]. Main reasons were a high workload, time pressure (lack of time), and stress perception. German caregivers are facing the non-use of breaks due to overtime, time constraints, and shift work on a practically daily basis [64]. However, breaks are an important work resource that are not used enough in care work, even though they would help caregivers to maintain physical and mental health [65]. When German nurses (*n* = 1003, of which *n* = 685 nurses and *n* = 318 geriatric nurses) were interviewed regarding the reasons for constantly not taking breaks, strong time and performance pressure emerged as the biggest factor. This led to physical discomfort but also to emotional exhaustion and increased night-time sleep disturbances [65]. Other research shows similar conditions during the daily work of nurses [63,66]. Understaffing, due to, e.g., sick leave, led to more work according to our respondents, which encouraged break absenteeism. This is also evident in the literature [66]. Since outpatient caregivers spend their working time while driving and at patients’ homes, the extent to which German caregivers can recover during working time in the outpatient setting should be evaluated. The conditions of work organisation immanent to the activity pose a particular challenge. The time and performance pressures to which caregivers are exposed can make it difficult to comply with legally defined breaks [26]. Ultimately, the smokers from our study took their breaks more often than the non-smokers. This is in line with other study results from inpatient care [45,115].

The regeneration behaviour after work was particularly evident in the sleep behaviour and respectively in the quality of the interviewed outpatient caregivers from our study. Most of them slept fewer than 6 h at night. In most cases, shift work and type of the shift, the poorer health status of patients, or work-related stress were reasons for a negative influence on sleep behaviour. The influence of shift work and shift type on sleep behaviour and quantity is also known from studies on inpatient nurses and midwives, (e.g., [38,56,116,117]). In a study of 52 nurses, the poorest sleep quality was shown when working night shifts in general [38], whereas our interviewees emphasised the influence of early shifts on poor sleep quality. Sleep restriction due to shift work might even increase snacking behaviour [39,108], which was frequent among our respondents. Workplace violence was not mentioned by interviewed outpatient caregivers as an influencing factor of sleep quality, but it is prevalent in the outpatient care setting, and it causes a higher stress perception [50]. Perceived work-related stress has also been proven to lower sleep quality among nurses working in shifts [116,117].

### 4.2. Personal Health-Promoting Behaviours of Outpatient Caregivers

In the context of personal health-promoting behaviour during work, the outpatient caregivers interviewed emphasised back-friendly work, regular hand hygiene, and wearing PPE while working. Regular hand hygiene, i.e., washing or disinfecting hands, is already widespread among healthcare workers (see. [118]) and also internationally required of healthcare professionals by the World Health Organization and Patient Safety [118] to ensure employees’ and patients’ safety alike. A recent mixed-methods study examining knowledge, behaviour, and compliance concerning hand hygiene of nursing home nurses in Germany shows that the hand hygiene behaviour of nurses can be influenced by that of their managers. Nonetheless, apart from the individual qualification, only a few nurses seem to implement the recommended hand hygiene correctly. However, the provision or presence of hygiene products, such as disinfectants, increased hand hygiene [119]. With regard to back-friendly working practices, however, it can be said that many carers in Germany suffer from musculoskeletal complaints, so proper implementation seems to be almost non-existent cf. [120]. However, the Institution for Statutory Accident Insurance and Prevention in the Health and Welfare Services (BGW) has published accessible guidelines and tips related to back-friendly work in care [121,122]. The most likely path of infection in nursing is smear infection. Protective clothing is designed to prevent infectious excretions or body fluids from contaminating the work clothes worn underneath or the hands. Thus, wearing PPE can be interpreted as a health-promoting behaviour [123]. However, the right use of PPE in nursing requires the right knowledge of caregivers in any case (see [124]), as the awareness of it can enhance the practice [125].

Personal health-promoting behaviour in leisure time was reported by our interviewees in terms of exercise and maintaining a healthy diet. However, the majority of respondents highlighted decreased health-promoting behavioural patterns in general since working in the outpatient care setting. This could be explained by their working conditions, which can be straining (e.g., a high workload, understaffing, time pressure) [85]. This explanation is also reflected in recently published research findings among 129 cancer caregivers: 60% were less physically active and almost half reported a poorer diet [126].

### 4.3. Factors Influencing Outpatient Caregivers’ Health Behaviour

Additional factors were reported by outpatient caregivers interviewed that were perceived as impairing the implementation of health-promoting behaviours. In particular, time pressure and delays due to constant searching for parking spaces and lack of on-site support from colleagues were mentioned. Ultimately all of the factors led to an increased perception of stress in general. This is also shown by results from inpatient geriatric care, (e.g., [127,128,129]), or among inpatient nurses [130]. Individual coping strategies can reduce the impact of negative consequences of negative strain reactions, such as stress, by making use of personal resources and thus can encourage the practice of health-promoting behaviours [16]. In our study, many outpatient caregivers described self-calming, smoking, and snacking as their most frequent coping strategies. Self-calmness and composure were also described as the primary coping strategy in a study among geriatric caregivers by Gutsch et al. [129]. Smoking as a coping mechanism is also reflected in other research results from inpatient care [131,132]. Perceived work-related stress might encourage an unfavourable way of eating, such as more processed foods or snacks, as shown among hospital nurses as well [37,103,133]. In private, some outpatient caregivers of our study talked about using progressive muscle relaxation training, which is also shown to improve sleep quality, in addition to reducing depressive symptoms [134]. Social support (work-side and private) and communication were also highlighted by interviewees as important resources to better cope with work-related stress perception. Other studies from the care setting underline our findings in this context [131,135,136,137,138,139].

### 4.4. Strengths and Limitations

A strength of this study is that we were able to recruit several outpatient caregivers from different care services of different city districts from Hamburg, Germany. Participants had a variety of socio-demographic characteristics, e.g., differences in age, length of work experience as well as working hours per week. Recruitment took place in a short period of time. Ultimately, we were able to establish a broad picture of health behaviour patterns of German outpatient caregivers, which has not yet been examined. To increase the trustworthiness of our findings, we employed rich descriptions of our results and displayed many direct quotes from the interviewees [140]. Furthermore, research results were profusely discussed in a group of researchers and were also contrasted constantly with empirical references.

However, there are limitations which need to be noted. Firstly, our findings are not based on a random sample. Since participants were partly chosen via snowballing technique an increased risk of self-selection cannot be excluded. For example, people who are more interested in one topic are more likely to participate in a study. Moreover, only three of our interviewees were male. However, female nurses tend to participate in studies more often than male nurses [141,142,143]. In addition, there are more female than male employees in care occupations in Germany [144].

At this point, it must be emphasised that part of the interviews were conducted during the early outbreak of the COVID-19 pandemic [145]. This may have had some impact on the results of the interviews, especially since, for instance, increased stress perception and extra work due to the pandemic was present among outpatient caregivers [16,146]. A further methodological limitation could be seen in the combination of face-to-face interviews and one-to-one telephone interviews. Although telephone interviews presuppose a certain relationship between interviewer and interviewee, it should be noted that there is no eye contact. Moreover, telephone interviews can lead to a distanced atmosphere [147]. In addition, telephone interviews might decrease possible social clues as well as promote the appearance of an asynchronous communication [148,149]. Although positive outcomes in the interview atmosphere (recognition of facial expressions, etc.) have already been reported via video telephony in qualitative research [150,151], all participants nevertheless spoke out in favour of a telephone interview due to the subjectively perceived reduced effort.

Another limitation can be the relatively small sample size. The results therefore must be reviewed in terms of transferability or generalisation [94,152]. All things considered, due to the nature of our qualitative research design, a generalisation of our results is impeded. However, individual statements of interviews can be significant [152]. Additionally, data saturation seemed to be achieved since this mostly occurred within the first twelve interviews [96]. Nevertheless, the results of the present qualitative study should be verified by studies with larger samples and especially by quantitative studies or in mixed-methods studies that could provide broader knowledge on the topic.

### 4.5. Implications for Further Research and Practice

Since outpatient caregivers represent a special group of employees, as they are always on the run to their patients, further research studies with larger sample sizes are needed. Especially since it is assumed that because of their special work activity in the field, the conduct of healthy behavioural patterns might be hindered [26]. In such studies it would be interesting to conduct further interviews with outpatient caregivers who have worked during the COVID-19 pandemic, inquiring about how their health behaviours might have changed over the time. In particular outpatient caregivers from Germany faced new challenges in their work activities due to the COVID-19 pandemic, which led to higher levels of stress [146]. Furthermore, it could be a future research desideratum, along with expanding the study sample size. A quantitative questionnaire study should be conducted to achieve a more representative study sample, e.g., characteristics which should be considered could be different ages and an even gender distribution. Part-time and full-time workers should also be differentiated by analysing and comparing their individual health behaviours. After such research has been carried out, specific interventions within the framework of work-side health promotion and occupational health and safety could be developed and implemented, as they could have a positive effect on outpatient caregivers’ health behaviour cf. [26,84].

Implications for further practice could be divided into two sections. On the one hand, behavioural (e.g., improving coping competences and/or education) measures could be carried out, and on the other hand, structural (e.g., changes in the work organisation and environment) prevention measures could be carried out [153,154,155].

Sufficient nutrition and hydration ensure that the body’s functionality remains unimpaired [156]. Outpatient caregivers should be educated about a healthy diet and nutrition (see [155]), as a higher health literacy could imply better health behaviour by implementing knowledge in practice [157]. For instance, the World Health Organization [158] has provided the most important facts about a healthy diet. Moreover, recent results from inpatient care show that higher resilience can also indicate better nutritional practices [159]. Educating outpatient caregivers about the negative effects of excessive caffeine (e.g., increased anxiety, impaired sleep) [110] and tobacco (e.g., increased risk of cancer) [160] use may help them to better control the amounts consumed (see [161]). Educating outpatient caregivers in terms physical activity in addition to nutrition is also advised (see [25,155]). On the behavioural level, it also might be sensible to educate and inform outpatient caregivers about the relevance of taking breaks and how it can positively influence their individual health and productivity [29,30,162]. In terms of regeneration after work, regeneration techniques could be taught, such as meditation or yoga [31,163,164]. Since impaired sleep quality might occur due to shift work [48] as well as due to occupation-related perceived stress [165], there is great potential in strengthening personal resources. Outpatient caregivers’ motivation regarding health behaviour should be strengthened in general in the future in order to achieve better implementation of behavioural patterns in general (see [166]). Furthermore, in order to enhance outpatient caregivers’ general resilience for decreasing perceptions of stress, trainings to strengthen personal resources are recommended [155]. Strengthening personal resources can have a positive influence on mental and physical well-being and can counteract the development of stress perception [129]. In view of the increasing number of days of incapacity to work among German caregivers due to musculoskeletal complaints [120], training outpatient caregivers to work in a way that is easy on the back can be indispensable. Especially given the fact that there seems to be a connection between stress perception and musculoskeletal disorders: they favour each other [167,168,169]. However, behavioural interventions seem to be more difficult to adopt than structural prevention measures (see [170]). Therefore it is quite important to focus on structural prevention and to eliminate possible sources that could prevent health-promoting behaviour at their origin [171].

On the structural level it is also important to create eating possibilities for outpatient caregivers (see [172]). Since break rooms and canteens seem to promote regular eating breaks (see [40,173]), employers should therefore create spatial possibilities for outpatient caregivers even if they are working in a mobile setting.

In the future, efforts should be made to introduce higher-level health-promoting processes in outpatient care as well, which could facilitate healthy eating (see [105]). Since people usually want to make food choices as convenient as possible [102], it can be assumed that healthy food choices are especially more difficult [26] in the mobile setting in which outpatient caregivers work, a setting that entails time constraints and hectic schedules [32]. Therefore, in the future, nutritionists should be available to advise in outpatient care practice. As a lack of toilet facilities could reduce fluid intake and job productivity (see [111]), employers in the outpatient care setting should ensure that their employees have sufficient access to toilet facilities during their work shifts, e.g., at patients’ homes (after consultation with them) or in particular public places that could only be used by outpatient caregivers. Fatigue and exhaustion resulting from increased workload and changing shift work should be avoided in the future. In this way, outpatient caregivers could have more energy and time to be physically active in their free time (see [48,52,57,103,104]). Overall, workplace health promotion offers, e.g., in the form of programmes to promote resilience, back-friendly work, and information sessions, e.g., on the topic of smoking, should be offered by the employer [174], or for instance, caregivers might be offered classes in yoga to decrease stress perception [164]. It is further of great relevance that employers not only understand the positive effects of regular breaks during (shift) work [30] but that they also need to schedule enough break times for each outpatient caregiver during their shift. Employers of outpatient care services should further always make sure that their employees follow their scheduled rest breaks [175]. Because finding a parking space is a burden for many outpatient caregivers, employers should strive harder to provide free parking for outpatient caregivers, e.g., via an application [176]. For instance, such a temporary special parking right (e.g., in no-stopping zones) had been introduced in parts of Germany for outpatient care services, but it was only valid until the end of June 2020 [177]. Accordingly, at the federal level, a special parking right for outpatient caregivers without a time limit should be introduced without restriction in the future upon proof of employment with an outpatient care service. Another possibility would be to have possible costs arising from (incorrect) parking (if there is no other way) covered by the employer in outpatient care. At the work organisation level, the implementation of a realistic tour with planned breaks should also be focused on [63,178]. Additionally, there should be steady shifts in consultation with employees in tour planning instead of constant shift changes [36]. Beyond that, employers in the outpatient care sector should place a greater emphasis on the health of their employees in general. The implementation of a company health management plan and the continuous realisation of preventive measures on behavioural and structural levels by professionals could be supportive [26].

In the future, attention should also be paid at the political level to the healthy working arrangements of people in caring professions, and their good health should be set as a widespread goal (see [179]), not neglecting to take into account the increasingly ageing employees, also visible in outpatient care [2]. All in all, healthy working conditions could not only encourage health-promoting behaviour of outpatient caregivers but also could improve their general health, motivation, and productivity [26]. Ultimately, caring for the caregivers is as important as caring for people in need (see [180]).

## 5. Conclusions

The present study focused on the health behaviour of outpatient caregivers, a yet unexplored field. The statements of the interviewees provide a highly differentiated insight into the individual experience and implementation of health behaviours of outpatient caregivers. Respondents in our study reported a variety of factors individually experienced in their working activity that had an impact on their individual health-related behaviours. Subjectively perceived barriers to adopting health-promoting behaviours illustrate the relevance of changing the way outpatient care work is organized in the future. Creating health-promoting working conditions and focusing on the personal empowerment of outpatient caregivers could help encourage health-promoting behaviours cf. [16,26]. Despite the limited number of interviews conducted, our results suggest that in the future particular occupational health and safety measures have to be implemented in order to promote healthy working conditions for outpatient caregivers. Finally, our results provide an adequate basis for developing specific health promotion measures at the behavioural and structural level.

## Figures and Tables

**Table 1:** Summary of current studies on nurses’ health behaviour.

First Author, Published Year, Reference	Study Design	Type of Health Behaviour	Study Population (*n*)
Najaf-Abadi 2018 [36]	Cross-sectional study	Health-promoting behaviour	Nursing staff (136)
Zapka 2009 [37]	Longitudinal study	Lifestyle behaviours	Hospital nurses (194)
Heath 2019 [38]	Cross-sectional study	Sleeping and eating behaviour	Shift working nurses (52)
Gifkins 2018 [39]	Interview study	Eating and drinking behaviour, sleep, and regeneration	Nurses (12)
Gupta 2019 [40]	Systematic review	Eating behaviour	Shift working nurses, 62 articles
Chiou 2014 [41]	Cross-sectional study	Eating behaviour and physical activity	Physicians (4202), hospital nurses (31,639), pharmacists (2315), other health professionals (8161) and administrative personnel (13,079)
Han 2012 [42]	Cross-sectional study	Smoking behaviour	Nurses (1724)
Silva-Costa 2012 [43]	Cross-sectional study	Sleeping behaviour	Nursing professionals (1307)
Lehmann 2014 [44]	Comparison study	Eating behaviour, physical activity, smoking behaviour, and drinking behaviour (alcohol)	Nursing students (259)
Sarna 2009 [45]	Web-based survey	Smoking and break behaviour	Nurses (2589)
McDowall 2017 [46]	Cross-sectional study	Sleeping behaviour	Nurses (888)
Palermo 2015 [47]	Cross-sectional epidemiological study	Sleeping behaviour	Nurses (1940)
da Costa Fernandes 2013 [48]	Transversal study	Eating and drinking behaviour, physical activity, sleeping behaviour	Nurses (2279)
Perry 2015 [49]	Cross-sectional study	Eating behaviour, physical activity, drinking behaviour (alcohol)	Nurses (381)
Zhang 2018 [50]	Cross-sectional study	Sleeping behaviour	Nurses (1024)
Klainin-Yobas 2015 [51]	Descriptive correlational study	Physical activity	Nursing students (335)
Torquati 2016 [52]	Focus group study	Eating behaviour and physical activity	Nurses (17), 4 focus groups
Schneider 2018 [53]	Cross-sectional study	Eating behaviour, physical activity, smoking behaviour	Nurses (471) and other healthcare workers (18,349)
Edwards 2018 [54]	Census data study	Smoking behaviour	Nurses (39,126) and doctors (12,684)
Wirth 2016 [55]	Cross-sectional study	Eating behaviour, physical activity, and smoking behaviour	Geriatric nurses (130), general healthcare and nurses (142) and educators (82)
Dorrian 2017 [56]	Longitudinal study	Sleeping behaviour	Nurses (21) and midwives (41)
Peplonska 2014 [57]	Cross-sectional study	Physical activity	Nurses and midwives (725)

**Table 2:** Interview topic list.

Phase of the Interview	Contents
1 Information phase	Introduction: study information, confidentiality, informed consent
2 Warm-up phase	Qualifications, working activity
3 Main phase	Eating and drinking behaviour, physical activity, smoking behaviour Break behaviour Regeneration after work Stress, coping strategies, support
4 Final phase and end of the interview	Socio-demographics of the interviewees and farewell

**Table 3:** Participant characteristics.

ID	Gender ^1^	Age	Qualification	Occupation	Work Experience	Work Schedule	Nationality
1	m	64	Health and medical nurse	Outpatient caregiver	31 years	Full-time	German
2	f	41	Physician assistant	Outpatient caregiver	4 years	Part-Time	German
3	m	53	Geriatric nurse and paramedic	Nursing specialist for intensive care and ventilation, commissioner for hygiene, medicinal products, and medical devices	6 years	Full-time	German
4	f	28	Health and paediatric nurse	Outpatient caregiver	2 years	Full-time	German
5	f	43	Caregiver	Outpatient caregiver and head of a small team	6 years	Part-time	German
6	f	51	Caregiver	Outpatient geriatric nurse and office administrator in the health sector	23 years	Full-time	German
7	f	56	Physician assistant	Outpatient caregiver	3 years	Full-time	Polish
8	m	38	Geriatric nurse	Outpatient caregiver	5 years	Full-time	German-Moroccan
9	f	49	Caregiver	Outpatient caregiver and supervisor	6 years	Full-time	German
10	f	43	Storekeeper	Outpatient geriatric nurse	1.5 years	Full-time	German
11	f	48	Geriatric nurse	Outpatient geriatric nurse and palliative care specialist	25 years	Full-time	German
12	f	52	Interior decorator	Outpatient caregiver	16 years	Part-time	German
13	f	23	Geriatric nurse	Health and care assistant in the outpatient care	2 years	Full-time	German
14	f	21	Home and family care	Outpatient home and family caregiver	2 years	Full-time	Polish
15	f	67	Geriatric nurse	Outpatient geriatric nurse	14 years	Part-time	German

^1^*n* = 15; f = female, m = male.

## Data Availability

The data analysed during the current study are not publicly available due to German national data protection regulation. They are available on individual request from the corresponding author.

## Appendix A

**Table A1:** Interview guide.

**General Information: Education and Qualification**
What is your current profession? Where are you working? How long have you been working as an outpatient caregiver? What is your employment condition? Do you work in a multi-shift system?
**Work activity**
What is a day at work like? What are the tasks and routines? What is the relationship between "working on the patient" and being in the car/traffic when you look at a shift? Does your working environment (company, car, climate) have an influence on you? How many hours are your contractually agreed weekly working hours? How many hours do you actually work per week?
**Eating behaviour**
How would you describe your eating habits? What do you eat?
What unhealthy foods do you eat? (why, how much?)
Are/were there any perceived connections between your eating behaviour and your job? Are there certain influences or connections between your professional activity and your eating behaviour? (→How does this manifest itself?) Do you only eat before and/or after your shift?
Can you eat during your shift?
How can you eat during your shift? Where do you have the possibility to eat during your shift?
Does your eating behaviour change on stressful days?
Do you eat snacks during the shift? Are there differences in your eating behaviour during and outside your working hours? Do you feel that you can eat a meal regularly? Do you have a desire to follow a healthier diet?
**Drinking behaviour**
What beverages do you drink? Can you drink while working?
How much do you manage to drink?
**Physical activity**
How would you describe your physical activity during your working hours? Are you physically active outside of your working hours? Has your physical activity changed when you started working as an outpatient caregiver? Do you have a desire to become more physically active?
**Smoking behaviour**
Do you smoke? When do you feel the need to smoke during your shift? Do you perceive any connections between your work in outpatient care and your smoking behaviour? Do you intend to quit smoking?
**Break behaviour during work**
How often are you allowed/able to take a break during working hours? How do you spend your break during work? Do breaks get cancelled during a work shift?
**Regeneration after work**
How long do you sleep on average at night or before your shift?
To what extent does your activity have an influence on your sleep quality? Do you have the opportunity to recover after your work? What do you do to relax/recover?
**Personal health-promoting behaviour**
How would you describe your personal health-promoting behaviour during work?
How would you describe your personal health-promoting behaviour in private?
**Potential work-related factors influencing health behaviour**
What factors do you perceive as negative influencing your health behaviour? What factors do you perceive as positive influencing your health behaviour? To what extent do you perceive stress? Which coping strategies do you use? To what extent do you receive support?
**Socio-demographic data**
How old are you? What is your nationality? What professional qualification do you have?

## Appendix B

**Table A2:** Code system.

Deductive	Inductive
Socio-demographic Data	
General professional data o Occupation o Type of employment o Work experience o Weekly working hours	
Work activity	
Eating behaviour o Description of own eating habits o Food choice o Amount o Times of food intake o Eating behaviour in times of stress perception o Snack intake o Change in eating habits due to work activity o Desire for a healthier diet	Challenges for healthy eating Places of food intake
Drinking behaviour o Beverage choice o Amount o Times of liquid intake	Challenges for sufficient liquid intake Problems due to working conditions
Physical activity o Physical activity during work o Physical activity after work o Change in physical activity due to work o Desire for being more physical active	Challenges for being more physical activity
Smoking behaviour o Smoker o Need for smoke o Impact of work activity on smoking behaviour o Intent to quit smoking	Challenges for quitting due to work
Regeneration at work: break behaviour o Break frequency o Break design o Break cancelling	Factors influencing break uptake/cancellation
Regeneration after work o Sleep behaviour o Influence of occupation on sleep quality o Recreational opportunities after work	
Personal health-promoting behaviour o Personal health-promoting behaviour during work o Personal health-promoting behaviour after work	
Work-related factors, which could influence health behaviour	Negative influence Job demands (time pressure, workload, shift work) Stress perception Positive influence Personal resources Coping strategies Support (private, external)
